# Pregnancy and Multiple Sclerosis: An Update on the Disease Modifying Treatment Strategy and a Review of Pregnancy’s Impact on Disease Activity

**DOI:** 10.3390/medicina56020049

**Published:** 2020-01-21

**Authors:** Guoda Varytė, Jolita Zakarevičienė, Diana Ramašauskaitė, Dalia Laužikienė, Audronė Arlauskienė

**Affiliations:** 1 Faculty of Medicine, Vilnius University, 03101 Vilnius, Lithuania; 2 Clinic of Obstetrics and Gynaecology, Institute of Clinical Medicine, Faculty of Medicine, Vilnius University, 03101 Vilnius, Lithuania; jolita.zakareviciene@santa.lt (J.Z.); diana.ramasauskaite@santa.lt (D.R.); dalia.lauzikiene@santa.lt (D.L.); audrone.arlauskiene@santa.lt (A.A.)

**Keywords:** pregnancy, multiple sclerosis, relapses, breastfeeding, disease modifying therapy

## Abstract

Pregnancy rates are rapidly increasing among women of reproductive age diagnosed with multiple sclerosis (MS). Through pre-conception, pregnancy and post-partum periods, there is a need for disease control management, to decrease chances of MS relapses while avoiding potential risks to the mother and the fetus. However, pregnancy is not always compatible with the available highly effective MS treatments. This narrative review provides the aspects of pregnancy’s outcomes and the impact on disease activity, choices of anesthesia and the management of relapses during the pregnancy and breastfeeding period. Available disease modifying treatment is discussed in the article with new data supporting the strategy of continuing natalizumab after conception, as it is related to a decreased risk of MS relapses during the pregnancy and postpartum period.

## 1. Introduction

Multiple sclerosis (MS) is an autoimmune disease of the central nervous system, affecting the brain, spinal cord and optic nerves. MS presents in two clinical forms: relapsing multiple sclerosis, manifesting with inflammatory attacks causing deterioration of neurological symptoms, and progressive MS, defined as the constant worsening of neurological function [1]. The prevalence of MS in a healthy population is 1:1000, while for identical twins the risk is 1:4 when one twin has MS [2]. Infection with Epstein–Barr virus (EBV), vaccinations, lack of ultraviolet (UV) radiation, repeated exposure to organic solvents, physical and emotional stress are considered to be the major risk factors of MS for genetically predisposed individuals [3]. MS is more common for women (70%), in the last 30 years, the ratio of MS between adult women and men has grown from 2:1 to 3:1. Possible underlying causes for the gender disparity in MS include improved access to health care, later childbirth, hormonal replacement therapy, obesity and smoking. However, vitamin D deficiency is the most plausible theory of this disproportionate rise of MS among females [4]. According to the statistics, approximately for nine out of ten patients symptoms occurs before the age of 50 and one of three female patients become pregnant after the diagnosis of MS [5]. The increasing number of MS among women of reproductive age remains a clinical issue, as the variety of disease modifying therapies (DMTs) holds possible side effects for the fetus and the woman before or after conceiving. Through the pre-conception, pregnancy and post-partum periods there is a need for disease control management to decrease chances of MS relapses while avoiding the potential risks to the mother and the fetus [6].

This article reviews the literature on the available evidence concerning pregnancy’s impact on disease activity, issues before conceiving, available disease modifying treatment and the management of relapses during the pregnancy and breastfeeding period, pregnancy’s outcomes and choices of anesthesia during labor and delivery.

## 2. Pregnancy’s Impact on MS Disease Activity

The pregnancy in multiple sclerosis (PRIMS) study was the first prospective study including 254 patients with MS (269 pregnancies). Women were followed-up during pregnancies and one year postpartum. Research results concluded that the rate of MS relapses decreased through pregnancy, mostly during the last trimester, while relapse rates increased 3 months after delivery and were equal to pre-pregnancy relapse rates [7]. Estrogens and other sex hormones activate immunological transformation during pregnancy by shifting T helper cells to mostly Th2 (anti-inflammatory effect), instead of Th 1 (pro-inflammatory effect), while after the delivery immunomodulation is reversed [8]. Animal studies on experimental allergic encephalomyelitis (EAE) demonstrated the significance of sex hormones on EAE manifestation, prognosis and disease activeness; these results are important for the progression of treatment for MS, as the study revealed the anti-inflammatory and neuroprotective impact of estrogens (17-estradiol (E2) and estriol (E3)), progesterone and testosterone [5]. The concentration of these hormones rises continuously during pregnancy and maximizes in the last trimester, providing the highest relapse protection, although a very recent study analyzed pregnancy related relapses in 375 women with MS (466 pregnancies). Annualized relapse rates (ARR) decreased to 0.14–0.07 compared to 0.39 ARR prior to conception and remained reduced for 3 months after delivery (0.27), turning back to prior pregnancy rates (0.37) in 4–6 months postpartum. The authors did not report any increased disease activity during the postpartum period [9].

## 3. Pre-Pregnancy Issues

Patients planning pregnancy have various concerns about fertility, the possibility of transmitting MS to the child, the impact of drugs on pregnancy, pregnancy’s influence on MS activity, and the mother’s capability to breastfeed and raise the child [5]. Women can be reassured that MS is considered not to have an impact on the woman’s ability to conceive and carry a fetus to term, as well as MS diagnosis does not increase the rates of premature or stillbirth, birth defects, cesarean delivery or spontaneous abortions [10]. MS patients are considered to have the same ability to conceive as healthy people, however women with MS have fewer pregnancies before the first attacks of the disease [11]. There was a case-control study performed analyzing anti-Müllerian hormone (AMH) concentrations in patients with MS compared to healthy individuals. The level of AMH, as the ovarian reserve indicator, was significantly lower in patients with MS compared to the control group [12].

Between patients with MS, sexual dysfunction is not uncommon. In a meta-analysis, study 61% of men and 63% of women (*n* = 14,538 patients) reported sexual intercourse problems due to MS [13].

As infertility rates among patients are around 10%, women experiencing failures to conceive may go through assisted reproductive techniques (ARTs), e.g., in vitro fertilization (IVF) with success up to 39% in women group under 35 years. Women who underwent an IVF procedure and failed to conceive, might be in a higher risk group for clinical or MRI disease activity for 3 months [10]. The increased risk of relapses is linked to the usage of gonadotropin-releasing hormone (GnRH) agonists. GnRH upsurges the production of IL-8, IL-12, INF-γ and TGF-β, known as pro-inflammatory cytokines, increasing the concentration of vascular endothelial growth factor (VEGF) and chemokine CXCL-12, increasing the transportation of mononuclear cells through the blood-brain barrier. This mechanism may illustrate heightened rates of MS relapses during fertility treatment as VEGF and VEGF-enhancing factor expression are significant in CNS angiogenesis during MS progression. In MS patients’ blood serum, endothelin-1 and angiopoietin-2 concentrations are significantly elevated and are considered to increase the angiogenic reaction of VEGF [14]. However, considering the fundamental role of angiogenesis in the normal fetal development process, targeting VEGF could cause in severe congenital malformations [15]. Sudden changes of estrogen concentrations during pregnancy and after delivery or termination of MS drugs during fertility treatment also have an impact on MS relapses rates [5].

Besides using folic acid and prenatal vitamins, eliminating alcohol and smoking, all women trying to conceive should be counselled to take vitamin D supplements [16]. Various studies reported an existing link between MS activity and lower levels of serum vitamin D concentration, suggesting that vitamin D deficiency correlate with MS development and higher MRI and clinical disease activity [17]. In recent years there was a prospective case-controlled research study in the Finnish Maternity cohort analyzing first trimester mother’s serum vitamin D concentration correlations with the chances of developing MS in the offspring. As a result, pregnant women with vitamin D deficiency (<12.02 ng/mL) had a twice higher risk of MS in the offspring in comparison with the not deficient control group. Research suggests that chances of MS development in the offspring may be diminished by obtaining adequate vitamin D levels during pregnancy [18]. Patients with MS have various concerns about the risks of transmitting the disease to children. According to the North American Research Committee’s database on Multiple Sclerosis (NARCOMS), 34.5% of MS patients made the decision not to have children due to the risk of disease inheritance [19]. Heritability can be described by genetic risk factors responsible for the immune response. Carriers of the allele from the MHC class II HLA *DRB1* gene, HLA DRB1*15:01, hold a three times greater risk of developing MS [2]. Patients can be counselled that the risk of passing the disease to offspring increases from 0.13% (general population) to 2%–2.5%. The chance of genetic inheritance rises up to 30% in two cases: monozygotic twins or both parents with MS [10].

## 4. Disease Modifying Therapies During Pregnancy

Disease modifying treatment during pregnancy needs to be adjusted individually taking into consideration the patient’s priorities, age, severity of disability, clinical and MRI disease activity, the rate of relapses and the risk of continuing or terminating the treatment [5]. According to the US Food and Drug Administration (FDA), the majority of drugs registered to treat MS are labeled as class C, meaning data of adverse effect was obtained in animal reproduction studies; however, despite the possible risk, pregnant women may benefit from the drug, but there is not enough reliable evidence and well-controlled studies in humans [20]. It is worth noting that DMTs are not certified during pregnancy, with the exception of glatiramer acetate 20 mg/mL [21]. Recently there was an analysis performed evaluating pregnancy outcomes (*n* = 142 pregnancies) in women exposed to DMTs during the first 3 months of pregnancy or at the time of severe disease reactivation. The most frequently used drugs included interferon-β (35.2%), natalizumab (19.7%), fingolimod (16.9%) and dimethyl fumarate (3.5%). The results reported no substantial differences in delivery outcomes between the groups who were exposed and not-exposed, as preterm birth rates were 5% against 3.2%, abortion 10% against 11.3%, and no data of birth defects were obtained [22].

### 4.1. Natalizumab

Natalizumab is a IgG4 humanized monoclonal antibody against α4-integrin (cell adhesion molecule) [16]. It is considered that natalizumab does not enter the fetal circulation until 13–14 weeks of gestation when the placenta is formed, but it actively crosses the placenta’s barrier during the second and third trimesters [16,23]. Discontinuation of natalizumab severely increases relapse rate, which normally manifests 12–16 weeks after treatment termination. New data supporting the strategy of continuing natalizumab after the conception is from an Italian study, presented at the 35th Congress of the European Committee for Treatment and Research in Multiple Sclerosis (ECTRIMS) 2019. In the current study women with MS were divided into three groups by the time of the last infusion of natalizumab: group 1, before conception; group 2, first trimester; group 3, continued treatment after the first trimester. After analyzing pregnancy outcomes, treatment continuation with natalizumab during pregnancy resulted in a decreased risk of MS relapses compared to the group with interrupted treatment early in pregnancy or before conception [24]. Even though evidence of natalizumab continuation safety is limited, the Association of British Neurologists incorporated these recommendations in their guidelines, suggesting to cut off natalizumab at 34 weeks of gestation and to resume as soon as possible after the delivery. In order to prevent MS relapses natalizumab should be resumed within 8–12 weeks after the last dosage [23]. With reference to the Tysabri Pregnancy Exposure Registry, 355 pregnancy outcomes were analyzed after exposure to natalizumab 3 months before conceiving or during pregnancy, as a result birth defects or spontaneous abortions were similar to the general population [25]. In a previous one case-series study, mild to moderate thrombocytopenia and anemia were detected in 10 of 13 newborns, when natalizumab was prescribed in the third trimester of gestation. As well, natalizumab was detected in all newborns’ umbilical cord blood samples (*n* = 5) [26]. When natalizumab is taken at the time of pregnancy, it is mandatory to test fetuses for thrombocytopenia and anemia after the birth [27]. Natalizumab treatment could be maintained during pregnancy as it is a promising strategy. This approach is only possible when the benefits to the patient outweigh potential adverse effects to the fetus as more evidence supporting the safety of this strategy is needed.

### 4.2. Interferon-β and Glatiramer Acetate

There is no data for interferon β (INF- β) or glatiramer acetate (GA) teratogenicity in animal studies. INF- β/GA sizable macromolecules do not cross the placental barrier in any substantial amount or interact with hormonal contraception [16]. No human studies have confirmed decreased fertility rates in women and men or linked INF- β/GA use to higher rates of miscarriage or inborn defects. Currently there are no abortion indications if unexpected pregnancy appears during a course of INF- β/GA, as interferon therapy has been safely implemented in viral hepatitis, myeloproliferative disorders, and multiple myeloma during pregnancy [23,28]. The use of INF- β or GA is approved for women trying to conceive with a high risk of MS rebound [21]. Regularly, MS relapse rates reduce during pregnancy, and for some patient’s treatment with INF- β/GA may be suspended. In cases when treatment continuation is necessary, INF- β/GA should be maintained as long as possible as there is no proof of any harmful effect to the fetus. When INF- β/GA treatment is terminated at the time of pregnancy, relapse rate may increase during the first months after delivery as restarted treatment requires a few months to gain maximum effectiveness [23].

### 4.3. Fingolimod

During pregnancy fingolimod should not be taken due to its effect on the receptors responsible for vascular system formation during the sensitive period of embryogenesis. Taking into account that after the treatment two months is required for fingolimod to be eliminated from the body, during this period contraception should be maintained to avoid potential danger to the fetus [29]. The Fingolimod clinical program analyzed pregnancy outcomes after exposure to the drug at the time of conception or 6 weeks before. Of the total 66 pregnancies after the exposure to the drug, 28 live births were included, 20 elective terminations, four terminations due to fetal abnormalities, nine spontaneous abortions, two fetuses were born with malformations (one congenital unilateral posteromedial bowing of the tibia and one with acrania) [30].

### 4.4. Dimethyl Fumarate

At the highest dose tested in rats, data of increased lethalness, delayed ossification and sexual maturity, lower birth and testicular weight were obtained [31]. Although, in human studies, results of the pregnancy outcomes when exposed to dimethyl fumarate early in pregnancy did not indicated increased fetal abnormalities [32]. The drug does not require a washout period due to its short half-life (approximately 1 h). At this time, not enough research is carried out about dimethyl fumarate excretion in breast milk, so breastfeeding should be avoided [33]. Women should be aware that gastrointestinal symptoms may occur within the first weeks of treatment due to possible drug absorption interaction with oral hormonal contraception [23].

### 4.5. Teriflunomide

Teriflunomide is contraindicated during pregnancy (classified in FDA pregnancy risk category X) as doses below those clinically used were linked to teratogenicity and embryo deaths in various species animal studies. However, no teratogenicity was observed in a human study analyzing 45 pregnancy outcomes that occurred accidentally while taking leflunomide (teriflunomide’s precursor to treat rheumatoid arthritis) [34]. Even though teriflunomide’s half-life is 16–18 days it may remain in the system up to 2 years. Conception is not preferable until teriflunomide concentration is under 0.02 mg/mL. In cases of accidental conception patient would benefit from quick elimination protocol when cholestyramine or activated charcoal is administered for a few days to reduce teriflunomide concentration to 0.02 mg/mL. It should be taken into consideration that the drug is also detected in semen, therefore drug elimination should be applied to men. Women should not breastfeed as teriflunomide is excreted into the breast milk [16,33].

### 4.6. Ocrelizumab/Rituximab

Anti-CD20 monoclonal antibody therapy includes humanized ocrelizumab and chimeric rituximab. As the evidence of ocrelizumab safety during pregnancy is very insufficient, the greatest experience of anti-CD20 antibody treatment in MS comes from chimeric rituximab. Immunoglobulins cross the placenta and enter the fetal circulation. B-cell lymphocytopenia lasting up to 6 months after the delivery was observed in animal and human fetuses after being exposed to rituximab in-utero [35]. Although at least a 12-month period after the last injection of rituximab is recommended before conceiving, a study analyzing 90 live birth outcomes of women inadvertently conceiving during or less than 12 months after the treatment of rituximab. The study reported 22 premature births, one neonatal death after 6 weeks, 11 newborns with hematological changes (B-cell deficiency, neutropenia, thrombocytopenia, anemia, lymphopenia), four newborn’s infections and two inborn malformations [36]. To avoid potential harm to the newborn, women should be advised not to breastfeed during or 6 months after discontinuing the treatment, as in animal studies rituximab was detected in lactating cynomolgus monkey’s milk [35].

### 4.7. Alemtuzumab

Alemtuzumab is a humanized IgG1 monoclonal antibody used to treat relapsing-remitting MS. Alemtuzumab targets CD-52, resulting in the depletion of B and T lymphocytes. Animal studies report increased embryo lethality and reduced levels of B and T lymphocytes in offspring when pregnant mice were exposed to alemtuzumab during the period of organogenesis [37]. Even though the drug concentration is incalculable in the plasma 1 month after the last dose, women are advised not to conceive for at least 4 months after alemtuzumab discontinuation [16]. A study was performed to analyze pregnancy outcomes in women treated in the alemtuzumab clinical development program. Out of 167 pregnancies, 66% healthy live births, 22% spontaneous abortions, 11% elective abortions and 0.6% stillbirth were reported [38]. Patients should be aware that the risk of autoimmune thyroid disease remains increased for 4 years after completing alemtuzumab treatment [23]. This is essential in pregnancy, as antibodies against thyroid stimulating hormone receptors cross the placenta and may induce transient neonatal Graves disease. To avoid autoimmune thyroid disease for women and possible harmful effects to the fetus, thyroid function should be tested regularly [16]. Breastfeeding is not recommended during treatment or within 4 months after receiving the last dose, as alemtuzumab is detectable in lactating mice’s milk and no data is available from human studies [37].

## 5. Pregnancy Outcomes and Anesthesia Choice

It is generally considered that having MS does not influence the risk of obstetrical fetuses’ risk, and epidural anesthesia has no significant impact on the disease [29]. A retrospective cohort study was conducted by analyzing data from the British Columbia (BC) MS Clinics’ database and the BC Perinatal Database Registry about pregnancy outcomes and negative effects to the neonates in a group of pregnant women with (*n* = 2975) or without (*n* = 432) MS. In comparison to the control group, infants of women with MS did not vary much in gestational age or weight, and having MS was not linked to increased rates of vaginal delivery or Caesarean section [39]. A similar prospective study was performed in Finland evaluating pregnancy outcomes in MS. Researchers assessed that compared to a healthy control group, the risk for pregnancy complications for women with MS was not increased, although collected data demonstrated a greater need for artificial insemination (4.9% versus 0.9%) and assisted vaginal delivery was performed more frequently (16.4% versus 6.5%) [40]. Last year, two publications investigating pregnancy complications were published. Sarah C. MacDonald’s et al. research included 3875 pregnancies in MS patients and identified higher rates of infections during pregnancy and preterm delivery. Other pregnancy and delivery complications rates were proportionate to ones among pregnant women without MS [41]. A similar study performed by Maria K. Houtchens et al. included 2115 pregnancies in the group of women with MS. Compared to the control group, women with MS during pregnancy and postpartum were more likely to experience infections, preterm labor, cardiovascular and venereal diseases, hematological alterations, neurological problems, also the fetuses of mothers with MS had an increased risk for acquired damage during delivery and inborn abnormalities [42]. As there are no contraindications for vaginal delivery, when counseling pregnant women with severe disability and spasticity of the pelvis or legs during the first months of pregnancy, the Association of British Neurologists guidelines recommends directing patients to a neuro-physiotherapist to collaborate with the patient and obstetric specialists during the delivery. At the time of labor, spasticity can be regulated by administering benzodiazepines and/or epidural anesthesia. Pregnant women with MS and affected spinal cord or no sensation below T11 may not recognize the beginning of labor. Patients should be trained to identify other symptoms indicating the start of labor, such as gastrointestinal discomfort, flushing and backache [23].

Women with MS should be aware that after the delivery, disease activity may increase, but these relapses are not influenced by the anesthesia received during labor or delivery. Methods of neuraxial anesthesia (NA) including spinal, epidural or spinal-epidural do not increase disease activity and are considered safe to MS patients. The greatest concern was that local anesthetics may act on demyelinated areas causing severe disease attacks or increase the rate of relapses [43]. Afterwards, several studies analyzing neuraxial anesthesia’s impact on MS declined previous concerns. The Italian cohort study (*n* = 423 pregnancies) aimed to evaluate cesarean delivery and epidural anesthesia’s effect on disability and relapse rate after the delivery. Authors concluded that epidural anesthesia can be safely performed as results did not link epidural anesthesia or cesarean delivery to increased disability or post-partum relapses [44]. A very recent Czech 10 year retrospective study (*n* = 70 pregnancies) reported no significant differences between cesarean or vaginal delivery and the choice of obstetric pain management did not influence MS activity 6 months after delivery [45].

## 6. Management of Relapses During Pregnancy and Postpartum Period

Despite the substantial reduction of disease activity during pregnancy, for some patients severe relapses appear whilst being pregnant [33]. At this time there are no contraindications for performing magnetic resonance imaging (MRI) during pregnancy up to 3 T, although contrast material should be avoided [16,23]. Gadolinium administered intravenously enters fetal circulation by crossing the placental barrier. Contrast material is secreted into the amniotic fluid through the urinary tract of the fetus. The risk of gadolinium dissociation from its chelate and turning highly toxic increases gradually with the time whilst the contrast agent remains in the amniotic fluid [46]. To avoid possible side effects for the fetus, low field strength (1.5 T) MRI without gadolinium should be performed in cases where MS relapse is suspected [47].

For the most part, for patients who are not pregnant or breastfeeding, MS relapses are treated by administering methylprednisolone intravenously or its analog orally [6]. Following the FDA pregnancy risk classification, corticosteroids are labeled in the pregnancy risk category C. Use of corticosteroids during the first trimester of pregnancy should be avoided as these drugs have been linked to fetal malformations, such as orofacial clefts among women using corticosteroids early in pregnancy [48]. Safe administration of corticosteroids depends on the type, dose of the drug, duration of therapy and the stage of pregnancy [47]. Prednisone, prednisolone and methylprednisolone cross the placenta at minimum concentrations and are considered to be safe to the fetus when administered in the second and third trimesters. At the time of relapses, 1 g of intravenous methylprednisolone for 3–5 days is recommended [33].

The risk of disease relapse is considered to be elevated for the first 3 months after delivery and MS activity is believed to be equal to pre-pregnancy levels. This change may be explained by the sudden alterations of pregnancy hormones concentrations after delivery [10]. Corticosteroids and intravenous immunoglobulins (IVIg) can be administered to treat and prevent postpartum relapses in breastfeeding patients [16]. There was a study performed evaluating the impact of intravenous methylprednisolone (IVMP) administration after delivery on the prevention of MS reactivation postpartum. As a result, relapses appeared in 17.9% of women treated with IVMP compared to a 46.2% relapse rate in the control group [49].

All MS patients should be advised to breastfeed after delivery, although there are uncertainties whether exclusive breastfeeding has an impact on disease activity postpartum. A prospective study was performed comparing MS patients exclusively breastfeeding 2 months after delivery with a group of patients breastfeeding for the same period not exclusively (partially or not breastfeeding). After a 1 year follow-up, the 38.3% not breastfeeding and 24.2% exclusively breastfeeding mothers experienced relapse within 6 months after delivery. As a conclusion, the authors suggested the opinion that women should be advised to breastfeed as it does not provoke relapses during the postpartum period [50]. A case report study, of MS breastfeeding patient receiving a 3-day course of 1 g methylprednisolone intravenously, reported that the drug concentrations in the infant blood samples were 0.207, 0.194, 0.164 mg/kg/day and the concentration peaked 1 h after breastfeeding. The obtained results were below the suggested dose (0.250 mg/kg/day) administered to infants when corticosteroid therapy is needed. The authors suggested that in cases where a short steroid treatment is given, breastfeeding should be considered safe, as the neonate’s exposure to the drug would be low [51]. Generally breastfeeding is not contraindicated in the background of methylprednisolone boluses treatment, but at least a 4 h interruption between drug infusion and breastfeeding is required [29].

## 7. Conclusions

Multiple sclerosis is considered not to have an impact on women’s ability to conceive and carry fetus’ to term, and as MS diagnosis does not increase rates of premature or stillbirth, birth defects, cesarean delivery or spontaneous abortions. Pregnancy substantially reduces disease activity, although for some patients, severe relapses could appear whilst pregnant. As the majority of drugs registered to treat MS are not compatible with pregnancy, women with a severe risk of disease reactivation would benefit from the continuation of treatment with natalizumab, as the latest data on the use of natalizumab have demonstrated that continued treatment during pregnancy was related to a decreased risk of MS relapses during the pregnancy and postpartum period.
